# Chitosan for Gene Delivery and Orthopedic Tissue Engineering Applications

**DOI:** 10.3390/molecules18055611

**Published:** 2013-05-15

**Authors:** Rosanne Raftery, Fergal J. O’Brien, Sally-Ann Cryan

**Affiliations:** 1 Tissue Engineering Research Group, Department of Anatomy, Royal College of Surgeons in Ireland, Dublin 2, Ireland; 2 School of Pharmacy, Royal College of Surgeons in Ireland, Dublin 2, Ireland; 3 Trinity Centre for Bioengineering, Trinity College Dublin, Dublin 2, Ireland

**Keywords:** chitosan, gene therapy, pDNA, siRNA, tissue engineering, gene-activated matrices

## Abstract

Gene therapy involves the introduction of foreign genetic material into cells in order exert a therapeutic effect. The application of gene therapy to the field of orthopaedic tissue engineering is extremely promising as the controlled release of therapeutic proteins such as bone morphogenetic proteins have been shown to stimulate bone repair. However, there are a number of drawbacks associated with viral and synthetic non-viral gene delivery approaches. One natural polymer which has generated interest as a gene delivery vector is chitosan. Chitosan is biodegradable, biocompatible and non-toxic. Much of the appeal of chitosan is due to the presence of primary amine groups in its repeating units which become protonated in acidic conditions. This property makes it a promising candidate for non-viral gene delivery. Chitosan-based vectors have been shown to transfect a number of cell types including human embryonic kidney cells (HEK293) and human cervical cancer cells (HeLa). Aside from its use in gene delivery, chitosan possesses a range of properties that show promise in tissue engineering applications; it is biodegradable, biocompatible, has anti-bacterial activity, and, its cationic nature allows for electrostatic interaction with glycosaminoglycans and other proteoglycans. It can be used to make nano- and microparticles, sponges, gels, membranes and porous scaffolds. Chitosan has also been shown to enhance mineral deposition during osteogenic differentiation of MSCs *in vitro*. The purpose of this review is to critically discuss the use of chitosan as a gene delivery vector with emphasis on its application in orthopedic tissue engineering.

## 1. Introduction

Chitosan is a polymer that has been used extensively both in nucleic acid delivery and tissue engineering applications. This review will discuss recent progress in these fields as well as the potential of combining both therapies into what has been termed, a gene-activated matrix (GAM) with a specific focus on orthopaedic tissue engineering. Gene therapy and tissue engineering are fields that have expanded immensely in the past three decades. Gene therapy was first conceived in the 1970’s as knowledge of the human genome grew exponentially [1]. Replacement of defective or missing genes to cure monogenic disorders such as X-linked severe combined immunodeficiency (X-SCID), cystic fibrosis and Duchenne muscular dystrophy was one of the first avenues explored and it was hailed as the new miracle cure for inherited disease [2,3,4]. However, this now accounts for just 8% of clinical trials while the use of gene therapy to manipulate cell behaviour, such as overexpression of certain cytokines targeting cancer cells, is now the most common use for the technology [5]. Tissue engineering involves the use of a scaffold to replace defective or injured tissue. The scaffold can be seeded with cells native to the defect site and/or loaded with growth factors or other cytokines to further enhance healing. Many natural and synthetic materials have been used in orthopaedic tissue engineering either alone or in combination with growth factors or cytokines in an attempt to enhance their therapeutic efficacy [6,7,8]. However, problems associated with the high release rates of these proteins at the injury site have led to toxicity and off-target effects. The high cost of recombinant proteins is also a major issue in growth factor delivery [9]. The combination of gene therapy and tissue engineering has huge potential as it can circumvent the problem of bolus release of protein by facilitating controlled growth factor release over time and in physiologically safe amounts, thus enhancing the therapeutic effect of the construct [10].

## 2. Chitosan

**Figure 1:**
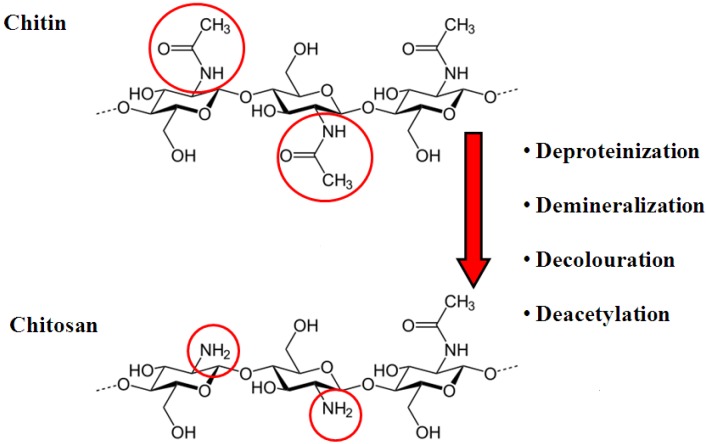
Molecular structures of chitin and chitosan [13]. Permission to reproduce the figure granted by authors and IOP publishing doi:10.1088/2043-6262/2/4/045004.

Chitin is a nitrogenous polysaccharide composed of β(1-4)–linked 2-acetamido-2-deoxy-β-D-glucose, most commonly derived from the exoskeleton of marine animals such as crab, shrimp, lobster and krill [11]. An estimated 2.3 million tonnes of chitin are produced as food industry waste each year making it an economical and renewable material [12]. However, chitin is considered chemically inert and is insoluble in water and organic solvents. *N-*deacetylation of chitin leads to its main derivative, chitosan (Figure 1). The degree of deacetylation is a major factor determining the characteristics of chitosan.

Chitosan is a natural linear cationic polysaccharide. It consists of two subunits, a D-glucosamine and an *N*-acetyl-D-glucosamine connected by (1-4) glycosidic bonds [11]. Each deacetylated group possesses three reactive functional groups—an amine group and primary and secondary hydroxyl groups [14]. The amine groups have a pKa value of 6.5, making chitosan a pH-responsive polymer [15]. At a pH of 7.4, the amine backbone of chitosan is neutral, while at a pH of 5.5, >90% of the amine groups are protonated, making chitosan soluble in organic acids [15]. The appeal of chitosan for nucleic acid delivery applications lies with the amine groups which, when positively charged, can bind to negatively charged molecules, such as nucleic acid, and form positively charged nano-sized complexes.

Being a natural polymer, chitosan possess a number of other favourable properties for use in nucleic acid delivery and tissue engineering applications. Chitosan is a biocompatible material, that is, it has been shown to interact with living cells without being cytotoxic or triggering an immune response while maintaining functionality *in vitro* and *in vivo*. Chitosan has an LD_50_ of 16 g/kg in rats making it equivalent to sucrose [16]. It has FDA approval for use as a wound dressing to accelerate healing and it also has approval as a lipid-binding food supplement in Japan, Italy and Finland [17] and has been designated as generally recognised as safe (GRAS) for use in food in the US [18]. Chitosan has also been shown to be biodegradable [15,19]. Chitinases, chitosanases and general lysozymes degrade chitosan into chitosan oligomers and monomers and finally into a common amino sugar, *N-*acetyl- glucosamine which then enters the glycoprotein cycle and is eventually excreted as carbon dioxide [20]. The rate of degradation of chitosan is related to the molecular weight and degree of deacetylation [19]. *N-*acetylglucosamine also has been found to have anti-inflammatory properties making chitosan suitable for *in vivo* applications [21]. Chitosan is also mucoadhesive making it highly suitable for gene delivery to epithelium including the lungs and gastrointestinal tract [20,22,23].

## 3. Gene Therapy

Gene therapy involves the introduction of exogenous genetic material into a target cell in order to exert a therapeutic effect. Viral or non-viral methods can be used to deliver nucleic acids into cells and their effect may be permanent or transient depending on the delivery method. Plasmid DNA (pDNA) is currently the most commonly investigated nucleic acid in gene delivery applications as it is cost effective, easy to expand and relatively non-toxic *in vivo* [24]. Upon gaining entry to the nucleus, the pDNA strand is transcribed and the coding gene is translated to protein which is then expressed from the cell. Another type of gene therapy is RNA interference (RNAi). RNAi is triggered by double stranded RNA (dsRNA) which activates the anti-viral interferon response leading to the shutdown of protein synthesis by degradation of messenger RNA (mRNA) [25]. Small interfering RNA (siRNA) consists of 21-23 nucleotides and can be designed to be more specific than long dsRNA and can avoid the activation of the interferon response while still inhibiting target gene expression [26]. Therefore siRNA transfected into mammalian cells can control gene expression in a highly specific way and has been used to block genes specific to certain infectious diseases and cancers. Another RNAi mechanism involves the use of microRNAs (miRNA) which are small non-coding nucleic acids responsible for post-translational regulation of protein expression [27]. Like siRNA, they are also short RNA sequences, usually approximately 19-25 nucleotides in length and bind to complementary (or almost complementary) sequences of target mRNA usually resulting in degradation of the mRNA and silencing of the gene being transcribed [28]. There are a number of methods used to modulate miRNAs that can be mimicked using siRNA-like technologies (preMirs) and inhibited using antisense oligonucletoide (antagomirs) [27]. Antagomirs are modified antisense oligonucleotides which contain a full or partial reverse complementary sequence of a mature miRNA. They can be used to block the effects of miRNA and therefore cause up-regulation of specific genes by relieving the inhibitory effect of the miRNA [27]. 

Over the past three decades, a number of gene delivery strategies have been developed. Nucleic acids can be delivered to cells either alone or within a viral [29] or non-viral [30,31] vector. Design of the delivery vector is hugely important as it must overcome a number of pharmaceutical and biopharmaceutical barriers depending on the nature of the nucleic acid and the cell type or *in vivo* target site. In order to deliver nucleic acids safely, the vector must confer protection from nucleases, reach the target cell, facilitate delivery across the target cell membrane, facilitate appropriate intracellular trafficking of the nucleic acid, and finally, in the case of pDNA, allow the nucleic acid cross the nuclear membrane and become transcribed [32]. Mature RNAi (siRNA and miRNA/antagomirs) do not need to enter the nucleus to cause an effect as these nucleic acids work in the cytoplasm. There are two main groups of delivery vectors; viral-based or non-viral-based.

### 3.1. Viral Vectors

Viruses have evolved over millions of years to become masters at entering cells and inputting their genetic information into the cellular genome [33]. Their innate highly efficient transduction ability is the reason why viral vectors are currently being used in approximately 70% of all gene therapy clinical trials [5,34]. However there are a number of concerns associated with their use including safety concerns and difficult manufacturing processes, the risk of toxicity and immunogenicity [35] and, most significantly, with integrating retroviral-based vectors, the possibility of insertional mutagenesis [36]. Other general issues include viral vectors inability to carry nucleic acid over a maximum of 34kB in size and the high costs associated with their development [35,37]. For some genetic disorders, viral vectors that can enable integration and long-term expression of the transgene is required, however, for other acute illnesses or tissue engineering applications, long-term expression is generally not necessary. While transient expression can be achieved using adenoviral vectors, their immunogenicity means that more and more researchers are turning to non-viral vectors for gene delivery [38].

### 3.2. Non-Viral Vectors

In contrast to viral vectors, non-viral delivery systems are less immunogenic, eliminate the risk of insertional mutagenesis, can carry large amounts of nucleic acid if required and are far more cost effective and safer to scale-up for manufacture [32]. However, the number of cells that successfully express or silence the desired protein, *i.e.*, transfection efficiency, reported using non-viral methods is inferior to that of viral vectors [39,40,41]. Non-viral delivery can be divided into physical or chemical methods. Examples of physical methods include electroporation [42,43] and direct injection of nucleic acid [44,45,46]. Electroporation is where an electrical pulse is applied to the cells to increase the permeability of the cell membrane facilitating uptake of DNA strands. Direct injection of pDNA has shown some success in muscle; however, without protection following systemic injection, the pDNA is rapidly broken down by nucleases [47]. Systemic delivery of siRNA, preMirs and antagomirs is even more difficult than pDNA as they are much less robust and degrade within 15 minutes in physiological environments [26]. Therefore, an appropriate delivery vector is required to exploit these therapeutics to their full potential. Chemical delivery methods, including cationic lipids and cationic polymers [32] are amongst the most commonly explored non-viral gene delivery methods. 

Lipid-based systems such as FuGene, GenePORTER, Transfast, DOTAP and Lipofectamine 2000^TM^ are commercially available lipid-based vectors. They are positively charged and encapsulate the anionic nucleic acid and enable cell entry via endocytosis. Lipofectamine 2000^TM^ is the most commonly used and often acts as a positive control in many experiments [48]. However, toxicity is a major side effect limiting their translation to the clinic [48]. Cationic polymers are positively charged materials which bind electrostatically to negatively charged nucleic acid to form delivery vectors [49]. Poly(L-lysine) (PLL) is a biodegradable cationic polypeptide capable of condensing pDNA into positively charged nanoparticles [50]. It has been used abundantly for both *in vitro* [51] and *in vivo* [52] gene delivery for over 30 years. PLL is fully protonated at physiological pH and therefore cannot cause endosomal release by buffering at low pH. Endosomal release has been improved with the inclusion of chloroquine, an endosomolytic reagent [53]. However, as PLL has been shown to be highly toxic to cells [54] and chloroquine cannot be used *in vitro*, PLL is rarely used for gene delivery. Another cationic polymer, polyethyleneimine (PEI), was first described as a gene delivery vehicle in 1995 [55] and since then has become the most popular non-viral gene delivery vector due to its high transfection efficiency [56,57,58]. PEI is a synthetic, water soluble cationic polymer that can be used in either its linear or branched forms. It has a wide ranging buffering capacity, binding DNA at a range of pH levels [57]. This buffering capacity also enables highly efficient endosomal release by attracting negatively charged ions into the endosome causing it to rupture and release the polyplexes into the cytoplasm – a mechanism called the “proton sponge” effect [59]. Again, PEI can be toxic to cells, a factor which is inhibiting its translation to the clinic [60]. Polyamidoamine (PAMAM) dendrimers also show promise as non-viral gene delivery vectors. They also bind to nucleic acids and enter cells via endocytosis [61]. Superfect^TM^ is a commercially available PAMAM dendrimer which is commonly used as a positive control in gene delivery studies [62]. The transfection efficiency seen with these vectors is less than that of Lipofectamine 2000^TM^ [63].

**Figure 2:**
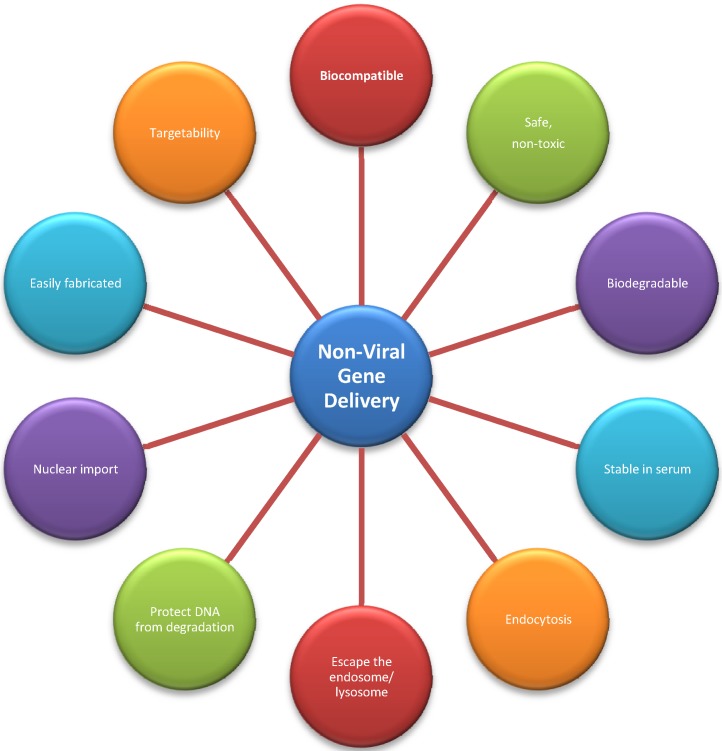
Non-viral gene delivery: The criteria to be met in the development of successful non-viral nucleic acid delivery vectors [32].

Another advantage of using polymer-based vectors is that they are open to modification; therefore, ligands can be conjugated to the polymer in order to target the vector to specific cell types [15]. The low transfection efficiency of non-viral vectors in comparison to viral vectors is due to the extra- and intracellular barriers that must be crossed to deliver nucleic acid to the nucleus; however, as mature miRNA and siRNA do not need to enter the nucleus to cause an effect, non-viral vectors may prove ideal for RNAi-mediated gene silencing. Pack and colleagues [32] summarized the criteria a non-viral vector must meet if it is to be successful in nucleic acid delivery (Figure 2). However, some cationic polymers exhibit high cytotoxicity due to their highly charged state which has limited their use clinically. Although complexation with nucleic acid can reduce theis charge, as these polymers are synthetic, there remain concerns regarding the ultimate fate of the construct and their degradation products [64]. Therefore there is a growing interest in the use of natural biocompatible and biodegradable polymers such as chitosan.

## 4. Chitosan and Gene Therapy

Mumper *et al.* were the first to report the use of chitosan for *in vitro* gene delivery [65]. As with all non-viral gene delivery vectors, chitosan-based vectors must meet the criteria outlined in Figure 2 [32]. These criteria include efficient cell uptake, protection of nucleic acids from nuclease degradation, escape from endolysosomal pathways, efficient unpacking of the nucleic acid cargo and nuclear import. A number of previous studies have shown that the physicochemical characteristics of chitosan complexes, *i.e.*, size, zeta potential (ZP) or charge and complexation efficiency with nucleic acid are crucial in overcoming physiological and cellular barriers to gene delivery [20,66]. It is generally accepted that complex diameter of <200 nm [48], carrying a positive zeta potential, is optimal for transfection. Key factors influencing both of these physicochemical properties and therefore transfection efficiency include the composition and preparation method of the chitosan nucleic acid formulations [67]. Chitosan was first used as an *in vitro* delivery vector for siRNA in 2006 [68]. Again the composition of the chitosan-siRNA complex and the formulation technique was important in achieving efficient gene silencing.

### 4.1. Methods of Preparation of Chitosan-Nucleic Acid Complexes

#### 4.1.1. Electrostatic Interaction

**Figure 3:**
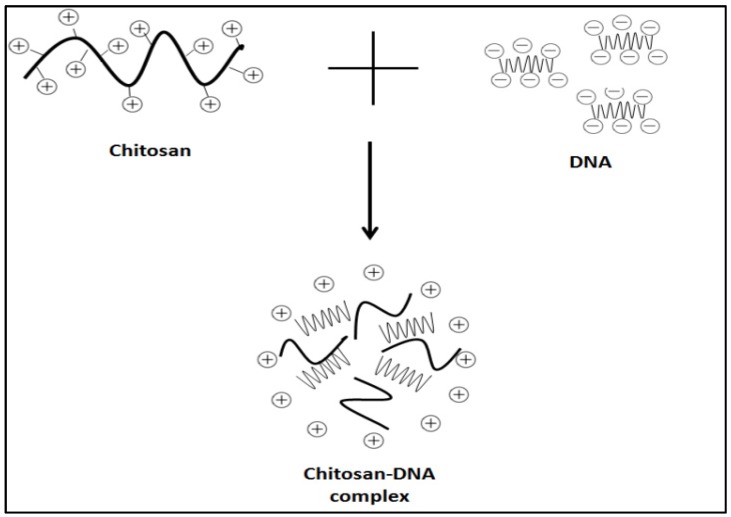
Electrostatic interaction between chitosan and DNA leads to the formation of a positively charged CS-DNA complex.

**Figure 4:**
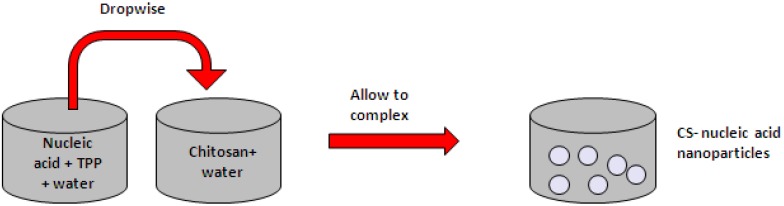
Schematic representation of ionic gelation method.

**Figure 5:**
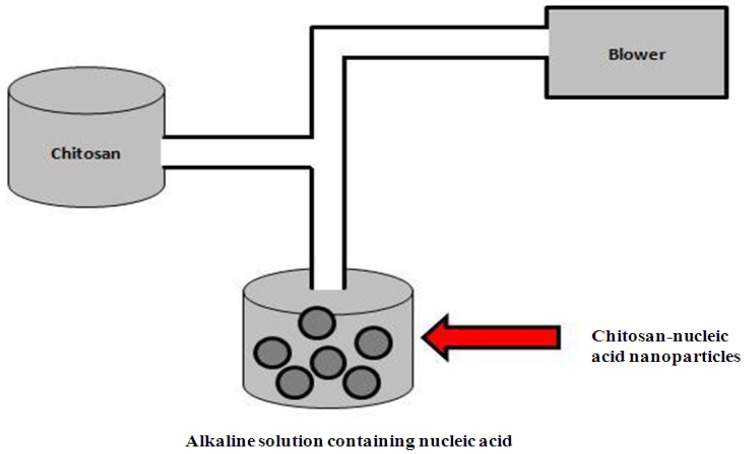
Schematic representation of complex coacervation formulation method.

The most basic method of forming chitosan-pDNA or chitosan-siRNA complexes is by direct mixing of positively charged chitosan with negatively charged nucleic acid, which then bind by electrostatic interaction (Figure 3) [67,69]. However, there are a number of different methods used to form spherical chitosan-pDNA nanoparticles. Two of the most commonly used methods are described below; ionic gelation (Figure 4) [70,71,72] and complex coacervation (Figure 5) [15,48,73].

#### 4.1.2. Ionic Gelation

The ionic gelation method is one of the most commonly used formulation methods in chitosan-based gene delivery as it is a very mild and straightforward process (Figure 4) [70,74,75]. It involves the use of a polyanionic cross-linker, usually tripolyphosphate (TPP). TPP cross-linking is a reversible procedure and has been shown to be biocompatible [76]. Chitosan is dissolved in an organic acid such as 1%–2% acetic acid and added dropwise to an aqueous suspension containing pDNA and TPP under constant stirring. Due to the electrostatic interaction between the oppositely charged ions, chitosan undergoes ionic gelation and chitosan-pDNA nanoparticles precipitate [77]. Particles formed using this method have a size distribution between 20 and 700 nm, depending on molecular weight of chitosan used [78,79,80,81]. They carry a positive zeta potential an d protect pDNA from DNase degradation [72]. Ionic gelation has also been used to formulate chitosan-siRNA nanoparticles where the siRNA was either entrapped within the nanoparticle or adsorbed onto the surface of a pre-made chitosan-TPP nanoparticle. Particles that contained entrapped siRNA led to more efficient gene silencing [68].

#### 4.1.3. Complex Coacervation

Complex coacervation is based on the spontaneous phase separation that occurs when two oppositely charged polyelectrolytes are mixed in an aqueous solution (Figure 5) [73]. Chitosan is insoluble at alkaline pH and precipitates when in contact with alkaline solution. Nanoparticles can be formulated by blowing a chitosan solution into an alkaline solution using a compressed air nozzle forming coacervate droplets [77]. The droplets are separated by filtration and centrifugation and are then washed. Mao *et al.* used this method to formulate chitosan-pDNA nanoparticles and formulated particles with a size range between 100 and 250 nm, a positive charge and partial protection from DNase degradation [15].

### 4.2. Mechanism of Chitosan-Nucleic Acid Complex Transfection

#### 4.2.1. Cell Uptake

**Figure 6:**
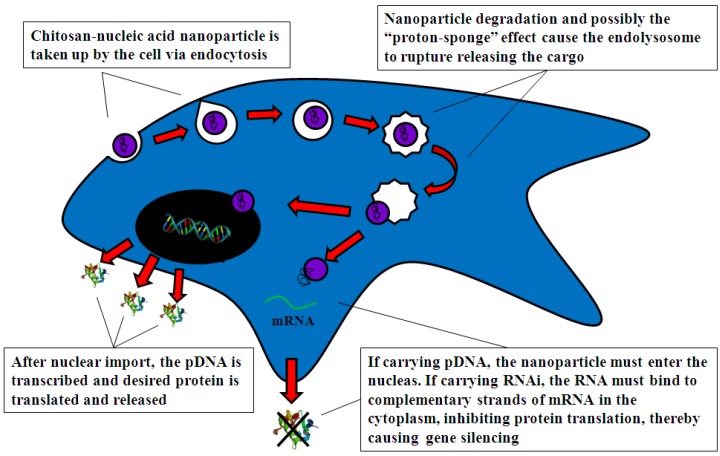
Schematic of chitosan-mediated transfection by endocytosis.

Polymer-based vectors can enter cells either by charge-mediated interactions with negatively charged proteoglycans on cell membranes or by receptor-mediated endocytosis [32] which is shown in Figure 6. Both processes result in the vector becoming trapped within an endosome and eventually a lysosome [31]. Cell uptake does not appear to be the rate limiting step in chitosan-mediated gene delivery once the particles carry a positive charge and have a diameter of less than 200 nm [48,82]. 

Some groups have conjugated ligands to chitosan-pDNA complexes in an effort to target the complexes to specific cells and increase cell uptake. The transferrin receptor is responsible for iron transport into cells and is found on many mammalian cells [15]. Transferrin can be bound to chitosan complexes via disulfide linkage. It is thought that by conjugating transferrin to chitosan, complexes will be able to enter cells via transferrin receptor mediated endocytosis which should, theoretically, increase cellular uptake [83,84]. Mao and colleagues used this approach to transfect human embryonic kidney cells (HEK293 cells) and a human cervical cancer cell line (HeLa cells). While a 3-fold higher transfection efficiency was reported with HEK293 cells, no increase in transgene expression was seen in HeLa cells [15]. This indicated that cell uptake is not the rate limiting step in chitosan-mediated transfection.

The KNOB protein is found on the adenovirus capsid [85] and, as the adenovirus is capable of transfecting a large number of cell types, researchers incorporated the KNOB protein onto chitosan complexes to investigate if an increase in transfection efficiency could be achieved. HEK 293 cells and HeLa cells were used in the experiment and KNOB conjugation improved transfection efficiency by 6–7 fold in HEK293 cells and 130 fold in HeLa cells [15].

In order to target chitosan-pDNA complexes to cells expressing a galactose-specific membrane, lectin, lactose was conjugated to chitosan and to the control vector, PEI [86,87]. Hep G2 and BNL CL2 cells, both liver cell lines, were then transfected with lactosylated chitosan. Transfection efficiency with lactosylated-chitosan-pDNA complexes remained poor however, lactosylated-PEI-pDNA complexes showed a 1,000-fold increase in gene expression. This indicated that the lack of efficiency is due to chitosan, not lack of receptor. More recently, galactosylated-chitosan was grafted with dextran and pDNA-containing complexes were prepared. Dextran was included in order to increase the stability of the complex. This system resulted in the efficient transfection of Chang liver cells [88]. PEGylation of chitosan–DNA complexes can be used to minimize aggregation by lowering the surface charge of the complexes, thus increasing cell uptake. It also allows for lyophilisation which can prolong stability in storage. Freeze-dried complexes retained their transfection efficiency after 1 month in storage [15].

#### 4.2.2. Intracellular Trafficking

Following endocytosis, chitosan-DNA complexes are held inside an endosome [89]. The pH of the endosome is approximately pH 5 and protons can enter the endosome through an ATPase-driven proton pump [90]. PEI is the most commonly used cationic polymer for gene delivery due to its wide ranging pH buffering capacity [20]. At pH 5 PEI is only partially protonated and can therefore attract negatively charged ions into the endosome causing it to swell and eventually rupture, releasing the PEI polyplex. This phenomenon is known as the ‘proton sponge’ effect [57,59,91]. At pH 5, chitosan is >90% protonated and it therefore exhibits a very weak ‘proton sponge’ effect and is subsequently much less efficient at escaping the endosome [15]. This is due to chitosan’s high pKa value of 6.5 and may indicate that chitosan does not act as a proton sponge, but rather degradation products increase osmolarity, which causes an influx of water and results in rupture of the endosome [60]. Intracellular trafficking of chitosan-pDNA complexes was compared to PEI-pDNA using confocal microscopy by Köpping Höggård and colleagues [20]. They found that at 24 h, PEI-pDNA complexes had already localized in the endosomal compartments and some were showing signs of damage and rupture. At the same time point, chitosan-pDNA complexes were also localized in the endosomes but no rupturing was seen until 72 h post-transfection. This later release corresponded to the delayed onset of gene expression seen with chitosan [20] and was supported by more recent work by Thibault and colleagues [82]. This suggests that endosomal escape is the major rate-limiting step in chitosan-mediated transfection. 

To improve the buffering capacity of chitosan and therefore cause earlier endosomal release, chitosan has been functionalized with imidazole moieties [92]. Functionalization did not affect chitosans ability to complex, condense and protect DNA from degradation and *in vitro* analysis on HEK293 and HepG2 cells showed improved transfection efficiency while maintaining cell viability. Chloroquine, an endosomolytic reagent has been incorporated into chitosan-pDNA complexes in order to enhance gene delivery [15]. It works by increasing the pH and causing disruption of the lysosome facilitating release of the delivery vector. However, no increase in transgene expression was reported [15]. Histidine is a non-essential amino acid which can increase the buffering capacity of some gene delivery vectors in endosomes and lysosomes. Chang *et al.* adapted this method for chitosan mediated gene delivery and found that it increased the buffering capacity of chitosan and subsequently, transfection efficiency was increased [93].

#### 4.2.3. Nuclear Import

Much less understood is how chitosan-nucleic acid complexes enter the cell nucleus. Non-viral gene delivery vectors as well as naked pDNA are all too large to enter the cell nucleus via nuclear pores. Subsequently, most complexes transfect cells undergoing cell division. The use of nuclear localization signal peptides can target pDNA to the nucleus and mediate entry [32]. On the other hand, mature siRNA and miRNA do not need to enter the nucleas to exert their effects and therefore nuclear import is not necessary for gene knockdown.

### 4.3. Factors Affecting Chitosan-Nucleic Acid Transfection Efficiency

It is generally accepted that non-viral nucleic acid delivery vectors should be less than 200 nm in diameter and carry a positive charge in order to facilitate cell uptake [48]. The vector should be stable enough to protect the cargo from degradation while also allowing for easy dissociation of the chitosan and nucleic acid to enable transfection. Molecular weight (Mw), degree of deacetylation (DD), ratio of chitosan to nucleic acid (N/P ratio), and nucleic acid concentration influence the eventual size and zeta potential as well as the nucleic acid condensation and stability of the vector.

#### 4.3.1. Molecular Weight

Chitosan’s molecular weight has a major influence on the eventual chitosan-nucleic acid complex size. MacLaughlin *et al.* reported that as the molecular weight decreased, so too did the chitosan-pDNA complex diameter [94]. This was unexpected, as it was thought that as molecular weight increased, the chitosan would condense plasmid more effectively. However, at high molecular weight (>150 kDa), chitosan is less soluble and more prone to aggregation than lower molecular weight chitosans [94]. This was supported by work completed by Huang *et al.* who showed that as the Mw decreased from 213 to 48 kDa, the complex diameter decreased from 180 to 155 nm. However, they found that as the molecular weight was decreased below 20 kDa, the complex diameter increased above 200 nm, indicating that complexes can be tailored to a specific size by varying the molecular weight [95]. High molecular weight chitosan has been reported to mediate higher levels of transgene expression than lower molecular weight chitosans [95,96,97]. This may be due to increased stability and protection from nuclease degradation. However, high molecular weight complexes exhibit a high degree of polydispersity and tend to form aggregates [22]. More groups have shown that low molecular weight chitosan mediates higher transfection efficiencies as chitosan dissociates more easily from pDNA leading to earlier and higher gene expression [98,99]. Low molecular weight chitosan complexes also tend to be smaller in size facilitating efficient cellular uptake and they also have more desirable pharmaceutical properties such as solubility at neutral pH and reduced viscosity. It is clear that a balance needs to be struck between stability and protection while also facilitating efficient pDNA unpacking and release. Köpping Höggård and colleagues sought to find the optimal low molecular weight chitosan that still retained the protective properties of high molecular weight chitosan. They found that an extremely low molecular weight chitosan consisting of 24 monomer units were stable as assessed by gel electrophoresis, capable of protecting pDNA from degradation, and facilitated higher gene expression levels than high molecular weight chitosan (approximately 400 kDa) and was comparable to PEI-mediated transfection efficiency in HEK293 cells [99].

As with pDNA, the Mw of chitosan has a big influence on chitosan-siRNA particle size, zeta potential, complexation efficiency and stability and thus, *in vitro* and *in vivo* gene silencing. Without protection, RNAi agents are rapidly broken down in physiological conditions; however, complete dissociation from a delivery vector is an essential step to allow for gene silencing. Therefore, the appropriate complexation/dissociation balance needs to be elucidated to achieve the optimal results. With pDNA delivery, low molecular weight chitosan has been shown to result in high transfection efficiency [99]; however, it has been reported that complexes formulated using chitosan with a molecular weight 64.8–170 kDa, the equivalent to 5 to 10 times that of the siRNA, results in stable complex formation and highly efficient gene silencing. However, at lower Mw, inadequate complexation is seen [100]. This indicates that what may work for pDNA delivery, may not necessarily be adequate for RNAi delivery. 

#### 4.3.2. Degree of Deacetylation

The degree of deacetylation (DD) refers to the percentage of positively charged acetyl groups on the chitosan backbone and therefore determines the charge of the polymer. Chitin is the inert non-acetylated parent polymer. Varying degrees of deacetylation lead to the formation of chitosans displaying various charge, solubility, crystallinity and degradation properties [101]. To obtain stable chitosan-pDNA complexes, Köpping Höggård *et al.* have reported that the DD should not exceed 65% [20]. Highly deacetylated chitosan (>80%) have resulted in reduced transfection efficiencies due to slow release of pDNA from the chitosan complex [96]. This is due to the high positive charge and therefore strong electrostatic interaction between chitosan and pDNA forming excessively stable complexes. Decreasing the DD may, therefore, reduce the strength of the electrostatic interaction and cause easier dissociation of pDNA from chitosan [98]. Decreased DD has also been shown to accelerate degradation [102,103]. Degradation of chitosan in the endosome may lead to earlier disruption and release of the vector which may lead to enhanced gene expression. Lavertu *et al.* found that using a low Mw and high DD chitosan, or, a high Mw with a low DD chitosan could enhance gene expression indicating the pivotal role the electrostatic binding plays in transfection kinetics using chitosan-based vectors [104]. For RNAi delivery, a DD of over 80% is required as this increases the charge of the complexes resulting in increased binding efficiency. Lower DD was shown to lead to the formation of unstable particles and low gene silencing efficiency [100].

#### 4.3.3. N/P Ratio

The N/P ratio refers to the ratio between the positively charged nitrogen (N) units on the chitosan polymer and the negatively charged phosphate (P) groups on nucleic acid. The overall charge of the chitosan-nucleic acid complex is determined by the N/P ratio and it also influences the ability of chitosan to fully condense nucleic acid which, in turn, affects the complex diameter [105]. An increase in N/P ratio means a greater amount of chitosan relative to pDNA which can enhance complex stability, lead to higher osmotic pressure in the endosomes thus enhancing pDNA release and subsequent transgene expression [20]. However, an N/P ratio that is too high can lead to excessively stable complexes that result in very slow or even no release of pDNA. N/P ratios that are too low lead to the formation of neutral or negatively charged complexes which cannot bind to the cell membrane and also tend to aggregate due to lack of inter-particular forces [106]. With siRNA delivery higher N/P ratios appear to induce more efficient gene silencing. In one study, N/P ratios of 50 and 150 were shown to enhance gene silencing in a human lung sarcoma cell line (H1299 cells) when compared to low N/P ratios of 2 and 10. The highest efficiency was seen at N/P 150 with 80% gene knockdown achieved. This result was due to the increased stability of these complexes at higher N/P ratios and the increased charge also facilitated enhanced cellular uptake [100]. This shows that the optimal N/P ratio is one that balances nucleic acid protection and stability with good release kinetics.

#### 4.3.4. Nucleic Acid Concentration

The pDNA loading dose it a critical parameter in transfection efficiency. Low doses of anionic nucleic acid lead to the formation of highly positively charge complexes which may be excessively stable and reduce transfection efficiency. In converse, high doses of pDNA may lead to the formation of charge neutral complexes which will encounter difficulty in cell uptake. Also, it has been shown that increasing pDNA dose causes an increase in complex size which may also affect cellular uptake [94]. With chitosan-mediated RNAi delivery, the ratio of RNAi to chitosan is extremely important in formulating particles in the optimal size and charge range. The RNAi ratio must be 5–10 times less than that of chitosan. 

#### 4.3.5. Nucleic Acid Dose

The transfection conditions also have an effect on transfection efficiency. The final dose of nucleic acid has been shown to effect transfection efficiency. One group have shown that increasing the pDNA dose from 0.5 to 2.5 μg/well led to increased transgene expression in epithelioma papulosum cyprinid cells, however, a further increase to 5 μg per well showed no further enhancement of transfection efficiency [107]. Supporting this finding, two other groups have shown that increasing the pDNA dose per well from 0 to 8 μg/well increased transfection efficiency in primary chondrocytes, however, further increases to 16 and 32 μg/well had a detrimental effect on transfection efficiency [108] and a pDNA dose of up to 2 μg/mL was optimal in SOJ cells with no further increase in transfection efficiency noted up to 10 μg/mL [89]. This may be due to inadequate charge on the complex carrying a large amount of negatively charged pDNA, aggregation of complexes due to reduced inter-particular forces, or an excessively large diameter restricting cellular entry. RNAi dose is even more critical as high doses can overwhelm the RNAi machinery of cells by blocking or increasing the expression of other miRNAs or causing cell death by RNAi-mediated toxicity [109,110]. 

#### 4.3.6. pH of Transfection Medium

As chitosan is a pH responsive polymer [66], it stands to reason that the pH of the transfection media would have an effect on transfection efficiency. At pH 7.4, chitosan’s amine groups have a neutral charge. However, at a pH of <5.5, over 90% of the amine groups are protonated, facilitating the electrostatic interaction between chitosan and pDNA forming small, stable positively charged complexes [15]. It has been reported that in a pH 7 solution, the zeta potential of chitosan-pDNA complexes is significantly reduced when compared to complexes in a pH 5 solution and neutrally charged complexes were found at a pH of 7.4 [15]. A number of other groups have also shown that a range of cell types transfected in media with a pH below 7 resulted in enhanced transfection efficiency when compared to physiological pH [104,108,111] These results are most likely due to reduced stability of chitosan-pDNA complexes at physiological pH leading to aggregation, neutralization of surface charge causing diminished cell interaction and early dissociation of the complexes [69,87]. However, *in vivo,* the pH cannot be controlled as in *in vitro* experiments, therefore more work needs to be done to optimize these vectors for *in vivo* gene delivery. The effect of pH on chitosan-RNAi transfection efficiency has not yet been fully elucidated. Most transfection studies have used media at a pH of 7.4 and up to 80% gene silencing has been reported [68,100,112,113]. In another study by Alameh *et al*. media at pH 6.5 was used and gene silencing of between 55 and 80% were reported among different cell types [114]. 

#### 4.3.7. Serum Content

Chitosan-pDNA complexes have been shown to be stable in serum for up to four hours [73]. This indicates that chitosan can protect nucleic acid from degradation until it reaches the endolysosome. Ishii *et al.*, found that transfection efficiency using chitosan-pDNA complexes was higher in the presence of 10%–20% serum but was dramatically reduced in serum content exceeding this and, interestingly, in serum free media [89] This was shown to be consistent in adenocarcinomic human alveolar basal epithelial cells (A549 cells] [111]. These results are thought to be due to the high viability of cells at these serum levels. In contrast, Lipofectin mediated transfection was greatly reduced in the presence of 10% serum and was completely inhibited by levels greater than 20% [111]. Chitosans resistance to serum inhibition is an exciting prospect as it suggests that chitosan-pDNA complexes will be efficient gene delivery vectors *in vivo.* siRNA is notoriously unstable and it has been shown to begin to degrade almost immediately in the presence of 5% serum and was fully degraded at 24h. However, when incorporated into chitosan-TPP nanoparticles, the siRNA is protected and not degraded fully until 72 h [68]. This result is supported by a study by Chen and colleagues where siRNA released from chitosan particles in a physiological environment retained its bioactivity [115].

#### 4.3.8. Stability of Chitosan-Nucleic Acid Complexes

Chitosan needs to protect pDNA from degradation *in vivo* until it reaches the nucleus. In the physiological environment, many factors challenge the stability of chitosan-pDNA complexes. Protection from DNase I or II is a routine experiment and depends on chitosans Mw, DD and N/P ratio. Köpping Höggård and colleagues assessed the stability of chitosan-pDNA complexes in excess salt (NaCl 3.5 M), detergent (SDS 0.5 M) and a polyanion, heparin. None of the conditions caused chitosan to release pDNA; however, when the complexes were incubated with chitosanase, a chitosan degrading enzyme, all of the pDNA was released from the complexes. Moreover, the released pDNA was not adversely affected by the complexation process [20]. 

#### 4.3.9. Toxicity of Chitosan Vectors

Chitosan is considered a non-toxic polymer and has an LD_50_ of 16 g/kg in rats, the equivalent of sucrose [16]. When compared to PEI *in vitro* on HEK293 cells, an MTT assay determined that at concentrations of 20 µg/mL or greater, PEI polyplexes were toxic with an IC_50_ of 75 µg/mL. Conversely, a dose of 630 µg/mL of chitosan was required to induce a cytotoxic response, corresponding to an IC_50_ of 0.5 mg/mL [20]. 

Regnström and colleagues performed a series of gene expression arrays in Balb/C mice which were administered pLuciferase delivered by either chitosan or PEI vectors to compare the toxicity profile at a genetic level [60]. The cyclooxygenases 1 and 2 (COX-1 and COX-2) are enzymes involved in prostaglandin synthesis and are expressed by cells during the inflammatory process. Both COX-1 and COX-2 expression was up-regulated in cells transfected with PEI but were down-regulated by chitosan. Another gene that was assessed was Heme-oxygenase-1 (HO-1) which is induced by oxidative stress, heat shock and inflammatory cytokines. HO-1 is up-regulated by PEI, indicating further adverse events; however, chitosan did not cause any up-regulation of HO-1. The mitogen-activated protein kinases (MAPKs) were also activated by PEI but not by chitosan. These results imply that an inflammatory response is initiated by PEI within 24h of transfection, an effect that is avoided by using chitosan. *N*-acetylglucosamine, a degradation product of chitosan is considered anti-inflammatory and may be responsible for this anti-inflammatory effect [21].

The same group then assessed the results of expression of these inflammatory genes on cell cycle and found that PEI also up-regulated genes associated with the p53 pathway. The p53 protein is involved in apoptosis indicating that PEI transfection can lead to cytotoxicity and cell death. Further, the p53 co-activator BRCA1 was highly expressed 72h post transfection which is a strong indication of cell cycle arrest. Chitosan again showed no significant increase in activation of the p53 pathway, further proving its innate biocompatibility [60]. 

#### 4.3.10. Modifications to Chitosan to Increase Transfection Efficiency

As chitosan is insoluble at neutral pH, this limits its use as a pharmaceutical delivery system. However, chitosan can be modified easily and a number of groups have attempted to improve its pharmaceutical characteristics. Chitosan oligomers, short chains of chitosan with a very low molecular weight, are readily prepared by the depolymerisation of chitosan in nitrous acid [94]. No side products are formed from this reaction and different lengths of oligomer can be separated by gel chromatography. Recent work has shown that chitosan oligomers are more suitable to gene delivery than high molecular weight polymeric chitosan [22,99]. This is due to its improved pharmaceutical properties including solubility in water and decreased viscosity. Chitosan oligomers composed of between 15 and 21 monomers were shown to be as efficient as PEI *in vivo* [99].

Another group have described the use of trimethylated chitosan where the primary amine groups in the C-2 position of chitosan are replaced with quaternary amino groups. By incorporating a quaternization degree of as low as 10%, solubility of chitosan was improved significantly and was soluble in acidic, neutral and basic solutions up to pH 9 [116]. Trimethylated chitosan oligomers (TMO) with degrees of quaternization of 40% (TMO-40) and 50% (TMO-50) were used in transfection experiments on fibroblast cell lines (COS-1 cells) and human intestinal epithelial cell lines (CaCo-2 cells). In COS-1 cells, both types of TMO chitosan increased transfection efficiency when compared to unmodified chitosan oligomers; TMO-50 caused a 5–52 fold increase depending on increasing N/P ratio from 6–14. TMO-40 was even better with efficiencies increasing from 26 to 131 fold along with increasing N/P ratio. Nevertheless, neither trimetylated nor unmodified chitosan were capable of transfecting highly differentiated CaCo-2 cells [117,118].

### 4.4. Cell Types Transfected By Chitosan Vectors *in Vitro*

Chitosan has been used to transfect a range of different cell types *in vitro.* As can be seen from the Table 1 below, transfection efficiencies are extremely variable among cell types. This can be related not only to differences in the composition and preparation of the chitosan-DNA/RNAi complexes and therefore the physicochemical properties of the complex (as outlined above) but also due to differences in cell surface receptors and charge of the cell resulting in mis-matched cell uptake. Intracellular differences such as varying levels of degradation enzymes may also affect intracellular trafficking and release of nucleic acid.

**Table 1:** Cell types that have been transfected using chitosan-pDNA complexes.

Chitosan-pDNA complexes *Cell Lines*							
Cell type	Origin	Mw	DNA content	+/− serum	pH of media	Transfection efficiency	Ref.
HEK293	Murine	390 kDa	0.1–5 µg/well	+	7.4	15%–20%	[73]
		390 kDa	0.1–5 µg/well	+	7.4	1%–18% (DNA dose dependent)	[15]
		160 kDa	0.33 µg/well	-	7.4	25%	[20]
		150, 400, 600 kDa	5–10 µg/well	+	7.4	10^4^pg βgal/mg protein	[48]
		10, 40, 80, 150 kDa	2.5 µg/well	+	6.5 & 7.1	0%–40% (Mw, DD, N/P, pH dependent)	[104]
		113 kDa	1 µg/well	+	7.4	25%	[72]
		4.7, 8, 11.6, 16.4, 24.8, 32.9, 146 kD	0.33 µg/well	-	7	5%–60% (Mw, N/P dependent)	[66]
A549	Human	52 kDa,	10 µg/well	+	6.9	10 × 10^4^ RLU	[111]
COS-1	Simian	7, 24, 32, 49, 74, 86, 92, 102, 230 and 540 kDa	10 µg/well	+ and −	7.4	+ serum: 1 × 10^6^ RLU/mg protein (102 kDa)	[94]
						−serum: 7.5 × 10^5^ RLU/mg protein (540 kDa)	
HeLa	Human	(1) 52 kDa	10 µg/well	+	6.9		[111]
		(2) 70 kDa	6 µg/well	+	7.4	10^6^–10^8^ RLU/mg protein	[87]
		(3) 390 kDa	0.1–5 µg/well	+	7.4	No transfection	[73]
***Primary cells***							
MG63	Human	150, 400, 600 kDa	5–10 µg/well	+	7.4	No transfection	[48]
MSCs	Human	150, 400, 600 kDa	5–10 µg/well	+	7.4	No transfection	[48]

HEK293 cells are the most commonly used cell type for chitosan-pDNA-mediated transfection and the highest transfection efficiencies were achieved with this cell type [15,20,48,66,72,73,104]. Of these, oligomeric chitosan with a pDNA dose of just 0.33 µg per well achieved an efficiency of almost 60% [66]. 

Corsi and colleagues studied chitosan transfection efficiency on three cell types, most notably MG63 cells (an osteoblast cell line) and mesenchymal stem cells (MSCs) which have been shown to readily differentiate into osteoblasts and lay down bone matrix [48]. MSCs are widely used in orthopaedic tissue engineering and transfection of these cells with genes encoding growth factors such as BMPs lead to bone formation both *in vitro* and *in vivo* [119]. While the results of this study showed very low transgene expression in MSCs, chitosan-DNA complexes were non-toxic to cells compared to Lipofectamine 2000^TM^ which caused a 40% loss in cell viability after 3 days [48]. 

**Table 2:** Cell types that have been transfected using chitosan-RNAi complexes.

Chitosan-RNAi ComplexesCell Lines							
Cell type	Origin	Mw	RNAi content	+/− serum	pH	Gene Silencing Efficiency	Ref.
CHO K1	Hamster	Chitosan hydrochloride 110 and 270 kDa; Chitosan Glutamate 160 and 470 kDa.	4 pmol/well (96 well plate)	+	7.4	Up to 82% with 470 kDa formulation	[68]
HEK 293	Human	Chitosan hydrochloride 110 and 270 kDa Chitosan Glutamate 160 and 470 kDa	4 pmol/well (96 well plate)	+	7.4	Up to 44% with 470 kDa formulation	[68]
H1299	Human	(1) 8.9–173 kDa (2) 114 kDa (3) 44–143 kDa	50 nmol/well (24 well plate); 50 nmol/well (24 well plate); 37.5, 75 and 150 nmol/well(96 well plate).	- + and − +	7.4 7.4 7.4	Up to 80% at high Mw 77.9% Up to 80%	[100] [112] [113]
HepG2	Human	11.8 kDa	10 pmol/well (96 well plate); 60 pmol/well (24 well plate).	+	6.5	55%	[114]
LS174T	Human	11.8–110.9 kDa	10 pmol/well (96 well plate); 60 pmol/well (24 well plate).	+	6.5	80% at low Mw	[114]
***Primary cells***							
Peritoneal Macrophages	Human	114 kDa	50, 100, 200 nmol/well (24 well plate)	-	7.4	86.9%	[112]

A number of groups have assessed the ability of chitosan particles in RNAi delivery and are summarized in Table 2. Chinese hamster ovary cells (CHO K1) and HEK293 cells were used to assess the gene silencing capabilities of chitosan-siRNA particles. 82% gene knockdown was seen in CHO K1 cells; however, gene silencing efficiency was much lower in HEK293 cells indicating that chitosan-siRNA mediated gene knockdown is highly cell specific [68]. In another study, H1299 cells, a naturally GFP-expressing human lung sarcoma derived cell line were treated with chitosan-siRNA particles targeted to knockdown GFP expression. Approximately 80% GFP knockdown was reported using chitosan-based delivery while the positive control, *Trans*IT-TKO (Mirus Corporation, Madison, WI, USA) showed 85% knockdown [100]. Peritoneal macrophages harvested from transgenic EGFP mice were used to assess chitosan-siRNA mediated gene silencing in primary cells. 89.3% GFP knockdown was reported within 24 h following a 4 h transfection period. Commercially available *Trans*IT-TKO was used as a positive control under the same conditions and no significant gene silencing was reported [112].

### 4.5. Chitosan Vectors *in Vivo*

Gene therapeutics can be delivered in a number of ways for clinical translation:

(1) *Ex vivo* transfection refers to the transfection of cells*in vitro* before applying them to the defect site or seeding them onto a scaffold and implanting that at the defect site. This method can involve extensive cell culture thus increasing the risk of cell contamination [120].
(2) Gene therapy complexes can be injected *in vivo* into the systemic circulation or implanted directly into a defect site. The theory is that endogenous cells can endocytose the complexes and become transfected. However, there is a risk of rapid excretion and off-target transfection [121]*.*

A novel approach to improve this method is to incorporate the complexes into a scaffold and apply that to the defect site [24,122]. In this way, the complexes are retained at the defect site and thus only exert an effect where required. The combination of scaffold and complexes in this way is termed a gene activated matrix and is discussed in detail in the Tissue Engineering Section below (Section 5).

Chitosan-pDNA complexes have been used in *in vivo* gene therapy studies, most commonly in the gastrointestinal tract and the lungs, thereby exploiting chitosans mucoadhesivness [123]. Using a murine model of peanut allergen-induced hypersensitivity, Roy and colleagues attempted to vaccinate the animals against hypersensitivity using chitosan-pDNA nanoparticles carrying the Arah2, an anaphylaxis inducing antigen [23]. Mice were immunized orally and as chitosan is a mucoadhesive polymer, chitosan-pDNA complexes adhere to the gastrointestinal epithelia and subsequently transfect these cells and immune cells located in the gut associated lymphoid tissue. The results indicated that mice vaccinated with the Arah2 gene before subsequent challenge with peanut extract had a significantly reduced risk of anaphylaxis after a single dose of nanoparticles. 

Following on from *in vitro* work by Köpping Höggård *et al.*, on HEK293 cells using low molecular weight chitosan (24-mer) [22], chitosan-pDNA polyplexes were delivered to mouse lungs via intra-tracheal injection [99]. Complexes formed at an N/P ratio of 60:1 mediated a 4-fold increase in luciferase expression compared to high molecular weight chitosan. Transfection efficiency increased 7-fold along with DNA dose from 5 µg to 25 µg. No immune response was noted after histological examination of the bronchiolar and alveolar regions. 

Recently, chitosan oligomers (Mw: 7.3 kDa; DD: >97%) have been used to deliver EGFP to the retinal cells to explore the potential of chitosan as a retinal gene delivery vector. *In vitro* work showed poor transfection efficiencies with human retinal epithelial cells; however, when the vectors were injected sub-retinally, the retinal pigment epithelial (RPE) layer and some photoreceptors became transfected and expressed EGFP. Following intra-vitreal injection, a more uniform dispersal of transfected cells were noted in the inner layers of the retina and the RPE layer. The authors state that this is an exciting development in research into gene therapy for macular degeneration and retinitis pigmentosa, leading causes of blindness worldwide [124]. 

Following intranasal administration of chitosan-siRNA particles in an EGFP transgenic mouse targeting EGFP, 43% knockdown was achieved after just one dose. These experiments highlight the potential of chitosan in RNAi delivery [112]. Chitosan-siRNA particles targeting tumor necrosis factor-α (TNF-α), a factor secreted by peritoneal macrophages involved in inflammation in rheumatoid arthritis, were use *in vivo* in a murine arthritis model. TNF-α knockdown efficiency of up to 44% was seen in peritoneal macrophages following intra-peritoneal administration after 2 hours [125].

## 5. Tissue Engineering

**Figure 7:**
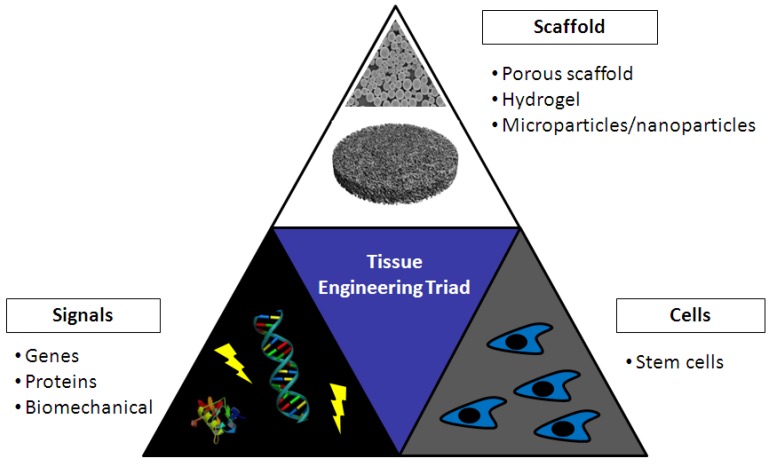
Schematic of tissue engineering triad.

Tissue engineering (TE) is a multifaceted discipline that combines the fields of cell and gene therapy with materials science and engineering, with a common goal to restore, replace, or enhance tissue function [126]. TE research is centered on the combination of three fundamental components, the tissue engineering triad: cells, scaffolds and signals (Figure 7). The scaffold provides a 3D support structure that attempts to mimic the natural extracellular matrix (ECM) for cells to infiltrate and expand. Signals can be either physical, in the form of a bioreactor [127], or biochemical, encompassing the addition of growth factors, genes or RNAi [24,58,128,129]. The purpose of the signals is to promote repair by the cells, attract endogenous cells to the site *in vivo* and encourage vascularisation and integration of the scaffold with the host tissue. 

The ideal scaffold should allow for cell attachment, proliferation, differentiation and extracellular matrix deposition. It must allow for neovascularisation to occur so that oxygen and nutrients can reach the centre of the scaffold and to facilitate the removal of toxins. The implanted construct should activate the innate healing mechanisms and it should degrade at a rate proportional to new tissue growth without the production of toxic by-products [126,130]. Typically scaffolds are fabricated from either natural or synthetic polymers, ceramics, or composite materials containing both polymer and ceramic components. There is an extensive list of polymers that have been assessed for use in bone tissue replacement, both of natural and synthetic origin which have been extensively reviewed elsewhere [6,131,132,133,134]. Natural polymers such as chitosan, alginate and hyaluronic acid as well as proteins such as collagen, fibrin and silk show promise due to their inherent biocompatibilities and biodegradabilities [135]. Many of these polymers also contain protonable functional groups which can be easily utilized in order to deliver bioactive factors such as proteins, drugs and nucleic acids [136,137,138]. The main limitations of natural polymers is their low mechanical strength and somewhat unpredictable degradation rate [135]. In contrast, synthetic polymers can be engineered to degrade at a tightly controlled rate and can exhibit mechanical and compressive strength similar to bone [134]. However, being synthetic, these polymers can elicit an immune response and the by-products of degradation are acidic which can lead to inflammation [64].

One of the most important characteristics of chitosan for tissue engineering applications is that it can be fabricated into structures of various forms [139]. Chitosan can form porous scaffolds with interconnected pores by lyophilization [19,140]. The interconnected porous structure is important as pores allow the infiltration of cells and vasculature throughout the construct. Chitosan can also form hydrogels suitable for tissue regeneration [141,142]. As chitosan is a pH responsive polymer, increasing the pH of dissolved chitosan solution can lead to gelation [143]. The addition of glycerol-phosphate salt to chitosan can form a thermoresponsive hydrogel which is liquid at room temperature but gels at body temperature [144]. This means it can be easily injected to the injury site. Due to chitosans powerful chelating ability, it easily forms complexes with metallic ions such as calcium (Ca), cobalt (Co), zinc (Zn) and nickel (Ni) [145]. As well as enhancing the anti-bacterial properties of chitosan (Co and Ni), these metal ions can improve the grafts strength and stiffness and enhance mineralization and vascularisation of chitosan scaffolds [146,147,148]. As described earlier, chitosan can also be used to formulate micro- and nanoparticles which can carry genes or therapeutic drugs to the injury site and exert an effect [48,70,77,99,123]. 

### 5.1. Chitosan in Bone Tissue Engineering

Bone is the primary structural component of the body, characterized by its rigidity and hardness. Bone is a particularly active tissue responsible for a wide range of functions and is capable of self-repair and remodelling [126]. However, when bone is injured beyond self-repair, a critical-sized defect, it can have life changing effects on the patient’s quality of life. Currently, the clinical gold standard solution to critical-sized defects is autografts, which is bone harvested from the patient’s own skeleton [149]. However, as there is a limited supply of bone that can be used in autografts, over 40% of patients cannot be treated in this way [150]. Thus allografts, which is bone donated from cadavers, are used. Immune rejection and disease transmission are big concerns regarding the use of allografts [151]. These limitations justify the development of new therapies for bone repair. Tissue engineering strategies might provide a viable alternative to current treatments. 

Scaffolds made from chitosan has proven a popular choice for bone repair applications since it has been shown to stimulate mineral deposition by osteoblasts [152,153]. Its cationic nature also allows for the electrostatic interaction with glycosaminoglycans (GAGs) and other proteoglycans, which is important as GAGs are known to control the actions of some cytokines and growth factors [139]. It promotes blood clotting after injury and has been shown to enhance the functions of inflammatory cells thereby promoting granulation [154]. Chitosan is degradable *in vivo* with the degree of deacetylation determining the rate of degradation—highly deacetylated chitosan degrades slowly and may last several months *in vivo* whereas chitosan with a lower degree of deacetylation degrades more rapidly [102,103]. As described previously, chitosan is also biocompatible [155], and has anti-bacterial activity [156]. 

Chitosan scaffolds are most commonly produced by lyophilisation. This involves freezing dissolved chitosan followed by drying which removes ice crystals leaving behind a porous structure. Tight control of temperature can dictate pore size. For cell infiltration and vascularisation to occur, pore size should be in the 100—150 µm range [139]. Electrospinning is another approach used in scaffold formulation producing a nanofibre mesh [157]. However, this method is considered quite difficult as the chitosan salt produced is soluble in water and therefore requires cross-linking which effects the pore structure of the mesh [158]. 

Muzzarelli and colleagues were the first to report the use of chitosan for *in vivo* bone regeneration. They implanted a chitosan ascorbate gel and chitosan membranes in cranial defects in cats [159]. They found that chitosan was exerting a stimulatory or attractive effect on stromal cells of the surrounding tissues. The same group went on to describe the use of methylpyrrolidone chitosan in critical-sized defects of the tibia in rabbits and the femoral head of sheep [160,161]. Again, a stimulatory effect on surrounding cells was reported. Methylpyrrolidinone chitosan was used to fill the space left in the mandible after wisdom tooth avulsion. The results showed that chitosan prompted osteoconduction and the formation of new bone which was mechanically and physiologically stable. The chitosan degraded over 6 months and no adverse effects were reported after one year [162]. 

The incorporation of chitosan with ceramics can improve the mechanical properties of the scaffolds. In one study, a chitosan gel reinforced with tricalcium phosphate showed compressive modulus much greater than that of chitosan alone. When implanted subcutaneously in a rabbit model, the above scaffold showed good biocompatibility while inducing a mild inflammatory response which receded as the scaffold degraded [163]. In another study, a chitosan nanohydroxyapatite scaffold was shown to significantly enhance osteoblast growth and extracellular matrix deposition as determined by an increase in osteocalcin production when compared to chitosan alone. This scaffold was tested *in vivo* in a rat calvarial defect and displayed good biocompatibility and osteoconductivity [164]. 

Chitosan based scaffolds can also be combined with growth factors to enhance their therapeutic efficacy. Stephen *et al.* evaluated the effects of a chitosan gel loaded with MSCs and BMP-2 in a rat calvarial defect [165]. Controls included the gel loaded with MSCs and the gel loaded with BMP-2. The combination of gel, cells and growth factor stimulated a significant increase in bone formation when compared to the control groups containing one or two of the above factors. Lee *et al.* described the production of a porous chitosan scaffold loaded with platelet derived growth factor-BB (PDGF-BB). This construct was assessed *in vivo* in a rat calvarial defect model and compared to an empty defect and a chitosan only scaffold. Osteogenesis was significantly enhanced in the chitosan scaffold defect when compared to the empty defect; however, complete union did not occur. The PDGF-BB eluting scaffold resulted in significantly increased bone formation when compared to both other groups and the authors described the chitosan scaffold as an excellent material for controlled release of protein [166]. De la Riva [167] described the creation of a brushite-chitosan composite scaffold that could release PDGF and VEGF at different rates mimicking the natural bone repair mechanism. Blank scaffolds, PDGF releasing scaffolds and PDGF-VEGF releasing scaffolds were implanted into a rabbit femoral defect and compared to empty defects. After 4 weeks, a higher degree of bone formation was seen in the dual growth factor eluting scaffold when compared to the empty, blank and PDGF alone scaffold indicating that delivery of more than one growth factor can have an additive effect on bone regeneration [167]. 

### 5.2. Chitosan in Cartilage Tissue Engineering

Cartilage, specifically hyaline cartilage, is an avascular tissue found on the articulating surfaces of joints. It facilitates the smooth movement of the joint and has a very limited capacity for repair. Damage to articular cartilage eventually leads to the development of osteoarthritis (OA] which is a degenerative disease that leads to the breakdown of the articular cartilage of the joint resulting in patients losing joint mobility and experiencing chronic pain [168]. OA is the most prevalent type of joint disease in the world affecting over one in six adults [169]. Treatment of OA includes debridement, microfracturing [170], and eventually total joint replacement [171]. Attempts have also been made to regenerate the joint using cell based therapies. An example is autologous chondrocyte implantation (ACI) which involves the implantation of autologous chondrocytes at the site of the defect [172]. Tissue engineering approaches for cartilage regeneration again includes the use of one or more of the components of the tissue engineering triad (Figure 7): scaffold, cells and signals.

The ideal scaffold for cartilage repair should closely mimic the articular cartilage matrix which is composed of type II collagen and GAGs [173]. GAGs are crucially important in chondrocyte viability and proliferation [174,175]. Chitosan consists of a variable number of *N*-acetylglucosamine groups which are also found in GAGs [16]. As GAGs are heavily involved in modulating chondrogenesis by interacting with growth factors and other cytokines, it stands to reason that chitosan may also possess related bioactivities. Chitosan has been shown to stimulate macrophages and attract neutrophils *in vitro* and *in vivo* [176,177]. These effects have been suggested to play a role in chitosan-mediated cell proliferation and integration of chitosan implants *in vivo* [178]*.* Chondrocytes are the ideal cell type to use, however, they are difficult to isolate and expand *in vitro*. MSCs have been shown to differentiate down the chondrogenic lineage and therefore may provide an alternative to chondrocytes [179]. 

In a study by Lu *et al.*, chitosan solution was injected directly into the articular cartilage of rat knees, which significantly enhanced chondrocyte proliferation [180]. This indicates that chitosan has a potentially beneficial effect on cartilage healing. A freeze dried chitosan sponge loaded with BMP-7 was assessed in medial femoral condyle defects in a rabbit model [181]. Histological analysis showed that the cells stimulating repair were of a chondrocyte phenotype; however, repair was not complete as the construct did not possess the required mechanical strength and was difficult to retain at the defect site. In an *in vitro* study, a porous collagen-chitosan-GAG scaffold was produced by freeze drying and loaded with chitosan microspheres containing TGF-β1. Chondrocytes were then seeded onto the scaffolds and cultured for three weeks [8]. The controlled release of TGF-β1 from the scaffolds promoted chondrocyte proliferation and significantly enhanced collagen type II and GAG production. This study demonstrates the versatility of chitosan as both a scaffold material and a delivery device for cartilage.

## 6. Gene Activated Matrices (GAMs)

Having discussed the use of chitosan as both a gene-delivery vector and a scaffold material for orthopedic repair, the final stage of this article will focus on the combination of gene therapy and tissue engineering therapies—gene-activated scaffolds or matrices (GAM), where chitosan has the potential to play an important role. While the release of proteins from scaffolds has shown enhanced tissue regeneration [10], recombinant proteins remain extremely expensive. The high burst release of protein can cause toxic effects and off-target responses such as ectopic bone formation seen with Medtronic’s’ INFUSE bone graft [9]. The inclusion of gene therapeutics within a scaffold affords the opportunity to provide sustained delivery of genes to specific cell types in a localized environment. Different combinations of genes and proteins can be released to create a microenvironment suitable for repair. It is proposed that seeded or endogenous cells migrate into the GAM, interact with and internalize the pDNA, and subsequently produce the desired protein [119]. Therefore, the GAM acts as a bioreactor and proteins can potentially be produced at natural levels for prolonged periods with careful design. Slower release of protein has been shown to lead to enhanced bone regeneration when compared to bolus release [10] which is seen with direct growth factor delivery. Incorporation of plasmid-encoding growth factors into a tissue engineering application also reduces the need for repeat doses of growth factors which can be toxic at excessively high levels [7]. Natural polymers such as chitosan and collagen possess intrinsic advantages over synthetic scaffolds as they tend to be more conductive to cell infiltration and proliferation [126]. The success of the GAM depends on cell infiltration as the more cells grow on the scaffold, the greater the level of cell uptake of the genes thus increasing the expression of protein. For this reason, design of the scaffold with adequate pore size and biocompatibility is extremely important. 

Bonadio *et al.* was the first to describe the use of gene-activated matrices. In that study, a collagen type I scaffold was used to deliver genes encoding a peptide fragment of human parathyroid hormone (hPTH 1-34) [24]. This GAM was used in the treatment of a 1 cm bone defect in a canine model. Union of the bone was seen after just 8 weeks with full healing seen after 53 weeks. In another study, also utilizing PTH embedded in a collagen scaffold, a 5 mm gap in rat femurs was filled with the GAM and displayed significantly increased mineralization compared to systemic delivery of hormone and local delivery of plasmid alone [182]. VEGF_165_ was also incorporated into a collagen sponge and shown to significantly enhance both vessel formation and bone repair in a rabbit radial diaphysis defect [183].

The addition of calcium phosphate particles to a scaffold is a popular choice in bone tissue engineering as calcium is a major component of bone [184] and the inclusion of these particles reinforces the mechanical properties of the scaffold [185]. *In vivo*, the calcium phosphate in bone is in the form of nano-sized hydroxyapatite (nHA) particles. Work within our group has shown that these particles are also efficient gene delivery vectors and contribute to enhanced calcium deposition both in 2D monolayer of MSCs and 3D collagen-based scaffolds [129]. Huang *et al.* assessed the transfection efficiency of GAMs containing PEI-pBMP-4 polyplexes in a rat cranial defect model *versus* an empty defect and a blank scaffold (no pDNA). A significantly higher amount of bone formation was found in the GAM when compared to the control groups as assessed by histomorphometry, micro-computed tomography and histology [186]. Within our group, PEI-pDNA polyplexes have been incorporated into a series of collagen based scaffolds to create a GAM for bone regeneration [58]. In a follow up study, PEI carrying a plasmid encoding Ephrin-B2 was assessed in 2D monolayer and on a 3D collagen-hydroxyapatite scaffold. The inclusion of PEI-Eph-B2 in a collagen hydroxyapatite scaffold, seeded with human MSCs, enhanced mineral deposition as early as 14 days. The GAM was capable of driving the MSCs down an osteogenic lineage which was the contributing factor to the enhanced mineralization reported [187]. 

Chitosan nanoparticles encoding PDGF a chitosan-collagen scaffold have been used in the *in vitro* assessment of a cell-seeded GAM for periodontal tissue engineering. Growth factor was released by the cells (periodontal ligament cells) for over six weeks and the cells retained their fibroblast-like shape and filled the pores of the scaffold. The cells laid down a periodontal tissue-like matrix [188]. The delivery of RNAi-based GAMs is a newly emerging area of research that has been reviewed elsewhere recently [189]. As chitosan can be used to deliver RNAi and as a scaffold, its use in RNAi based GAMs offers immense potential. 

The aim of this review was to give an overview of the use of chitosan in gene delivery and tissue engineering applications. One of the most exciting prospects for chitosan applications in the future is its application in advanced tissue engineering constructs, such as gene-activated matrices. As chitosan can carry nucleic acid and appears to be osteoinductive and chondro-inductive, its use in orthopaedic tissue engineering applications holds a lot of potential. MSCs are a popular cell choice for use in orthopaedic tissue engineering and chitosan appears to have a stimulatory effect on their proliferation both *in vitro* and *in vivo* [153]. Transfection of MSCs with chitosan-pDNA complexes was attempted by Corsi *et al.* but showed very low efficiency [48]. However, as conveyed in this review, there are a huge number of variables in chitosan-pDNA complex fabrication, thus enhanced transfection of MSCs may be achieved by optimization of a transfection protocol. Unpublished work within our group has shown that by comprehensively optimsing the composition and fabircation of chitosan-pDNA complexes, significant MSC transfection can be achieved. We have determined that chitosan-pDNA complexes (Mw 160 kDa, DD 80%, N/P 10) are capable of inducing transfection efficiencies in MSCs of up to 16.5% using reporter plasmids [green fluorescent protein (GFP)] with prolonged gene expression seen compared to PEI-mediated transfection. There was no toxicity seen in chitosan transfected cells; however, with PEI transfected cells there was significantly less live cells at 24 and 72 h as determined by MTT assay [190]. Work is ongoing to assess these particles ability to deliver therapeutic genes to MSCs and subsequently create a GAM for orthopaedic applications. *In vivo*, it is thought that MSCs are the cell type responsible for endogenous repair; therefore, the ability of chitosan-pDNA complexes to transfect MSCs is highly desirable. 

## 7. Conclusions

Chitosan has a number of key properties including; biocompatibility, biodegradability, anti-bacterial, -fungal and -viral properties, and a cationic nature that make it a useful material in gene delivery and tissue engineering applications. It can be fabricated into a range of architectures including nano- and microparticles, sponges, gels, membranes and porous scaffolds which can support a wide range of platform designs. It has been extensively explored for drug delivery applications including gene delivery. The development of a so-called “artificial virus” for non-viral gene delivery is still underway and chitosan’s biocompatibility and GRAS status have led to extensive exploration of this carrier for gene delivery including both pDNA and siRNA-based therapies. Limited transfection efficiency *in vitro* and *in vivo* initially hampered clinical and commerical translation of chitosan-based gene therapies. As outlined in this review a number of factors can be controlled to enhance transfection including chemical manipulation and fabrication factors. Key recent developments in composition including oligomeric forms and ligand-targeted constructs, along with a greater understanding of fabrication factors are leading to greater levels of transfection efficiency being seen both *in vitro* and *in vivo*. The purpose of this review is to critically discuss the use of chitosan as a gene delivery vector with emphasis on its application in tissue engineering. Chitosan as a scaffold material has shown promise in both bone and cartilage regeneration. It has been shown to stimulate mineral deposition by osteoblasts and to have advantageous immunomodulatory properties that may play a role in chitosan-mediated cell proliferation and integration of chitosan implants *in vivo*. Only limited work has been conducted to-date on its utility to deliver growth-factors from scaffolds, an obvious application of a material that has been so widely applied to drug delivery applications. Encapsulation of growth factors into chitosan-based nano- or micoparticulare systems to be loaded within scaffolds could offer significant control of growth factor release, with spatiotemporal control required for effective tissue engineering. Equally, the ability of chitosan to deliver pDNA to MSCs is being explored for application in gene-activated matrices and we have shown that by optimising the composition and fabrication of chitosan-pDNA complexes, successful and sustained transfection of MSCs can be achieved, which opens up this carrier as a vector for GAMs. The delivery of RNAi including siRNA and miRNA modulators (preMir and antagomirs) from implantable tissue engineering scaffolds to create RNAi-based GAMs is a newly emerging area of research that requires appropriate means of incorporating these nucleic acids into the scaffolds. Chitosan already has proven ability to transfect certain cell types with siRNA; now, together with its advantageous tissue engineering properties, its use in RNAi based GAMs offers immense potential.
